# Supplementary material for: Predicting hospital readmission risk: A prospective observational study to compare primary care providers’ assessments with the LACE readmission risk index
Source: PLoS One. 2021 Dec 15;16(12):e0260943. doi: 10.1371/journal.pone.0260943 (PMC8673665; doi:10.1371/journal.pone.0260943)
Supplement: S1 File — (DOCX) [file pone.0260943.s001.docx]

**Readmission Risk Assessment Survey**

Physician ID:

Having reviewed the discharge summary please complete the brief survey and place it in the box labelled “readmission risk study”. Many thanks for taking the time to complete this survey.

1. Has this patient been seen in clinic since discharge from hospital relating to the admission of the discharge summary?

Y/N

1. To your knowledge, has this patient been readmitted to hospital since discharge from hospital relating to the admission of the discharge summary?

Y/N

1. Having reviewed this discharge summary, how would you classify this patient’s risk for readmission?
   1. Low <10%
   2. Medium 11-20%
   3. High >20%
2. What factors helped you make this decision? (Please check all that apply)
   1. Age of patient
   2. Familiarity with patient history
   3. Admission diagnosis
   4. Medication list
   5. Co-morbidities
   6. Number of previous admissions in the last 2 years
3. Is this a patient you feel requires follow-up in your clinic?
   1. Yes
   2. No
4. What factors influenced your decision to follow-up? (Please check all that apply)
   1. Hospital admission diagnosis
   2. Familiarity with patient
   3. Known lack of social supports
   4. Patient co-morbidities
   5. Recommendation from hospital
   6. Other (please specify)___________________________
